# Circulating Prostate Cells Found in Men with Benign Prostate Disease Are P504S Negative: Clinical Implications

**DOI:** 10.1155/2013/165014

**Published:** 2013-04-17

**Authors:** Nigel P. Murray, Eduardo Reyes, Leonardo Badínez, Nelson Orellana, Cynthia Fuentealba, Ruben Olivares, José Porcell, Ricardo Dueñas

**Affiliations:** ^1^Division of Medicine, Hospital Carabineros of Chile, Simón Bolívar 2200, Ñuñoa, 7770199 Santiago, Chile; ^2^Instituto de BioOncología, Avenida Salvador 95, Oficina 95, Providencia, 7500710 Santiago, Chile; ^3^Circulating Tumor Cell Unit, Faculty of Medicine Universidad Mayor, Renato Sánchez 4369, Las Condes, 7550224 Santiago, Chile; ^4^Faculty of Medicine, Universidad Diego Portales, Manuel Rodriguez Sur 415, 8370179 Santiago, Chile; ^5^Urology Service, Hospital Carabineros of Chile, Simón Bolívar 2200, Ñuñoa, 7770199 Santiago, Chile; ^6^Radiotherapy, Fundación Arturo López Pérez, Rancagua 899, Providencia, 7500921 Santiago, Chile

## Abstract

*Introduction*. Developments in immunological and quantitative real-time PCR-based analysis have enabled the detection, enumeration, and characterization of circulating tumor cells (CTCs). It is assumed that the detection of CTCs is associated with cancer, based on the finding that CTCs can be detected in all major cancer and not in healthy subjects or those with benign disease. *Methods and Patients*. Consecutive men, with suspicion of prostate cancer, had blood samples taken before prostate biopsy; mononuclear cells were obtained using differential gel centrifugation and CPCs detecting using anti-PSA immunocytochemistry. Positive samples underwent further classification with anti-P504S. *Results*. 329 men underwent prostate biopsy; of these men 83 underwent a second biopsy and 44 a third one. Of those with a biopsy negative for cancer, 19/226 (8.4%) had CPCs PSA (+) P504S (−) detected at first biopsy, 6/74 (8.1%) at second biopsy, and 5/33 (15.2%) at third biopsy. Men with cancer-positive biopsies did not have PSA (+) P504S (−) CPCs detected. These benign cells were associated with chronic prostatitis. *Conclusions*. Patients with chronic prostatitis may have circulating prostate cells detected in blood, which do not express the enzyme P504S and should be thought of as benign in nature.

## 1. Introduction 

Although the first report on circulating tumors was published by Ashworth in 1869 [1], the lack of technology precluded further investigations on their clinical use until recently. Developments in immunological and quantitative real-time PCR-based analysis have enabled the detection, enumeration and characterization of circulating tumor cells (CTCs). The monitoring of CTCs has the potential to improve therapeutic management at an early stage and also to identify patients with increased risk of tumor progression or recurrence before the onset of clinically detected metastasis. Furthermore, the molecular profiling of CTCs can provide new insights into cancer biology and systemic treatment in neoadjuvant or adjuvant settings.

It is assumed that the detection of CTCs is associated with cancer, based on the finding that CTCs can be detected in all major cancers and not in healthy subjects or those with benign disease [2]. Most of the assays are based on enrichment and subsequent identification of CTCs using monoclonal antibodies directed against epithelial epitopes, for example, the transmembrane glycoprotein epithelial cell adhesion molecule (EpCAM) or cytokeratins that are expressed on both normal and malignant cells. This widely based approach is based on the fact that blood cells usually lack detectable expression of epithelial markers, being of mesenchymal origin. What remains to be confirmed is whether or not trafficking of normal epithelial cells could occur in certain benign conditions and might contribute to false positive findings in the current assay methods unless unambiguous criteria for the malignant nature of the marker positive cells are used.

However, although the number of epithelial cells in the blood of healthy individuals and patients with a variety of nonmalignant disease is low, they may be present in 0.3% of cases [2]. Furthermore, using the EpCAM-based CellSearch (Veridex) methodology, Pantel et al. [3] showed that circulating epithelial cells are found in between 0% and 18.7% of patients with benign colonic disease; the EPISPOT Cytokeratin 19 assay found circulating epithelial cells in between 8.3% and 28.6% of patients with benign colonic disease. Therefore, in patients's pretreatment, with suspicion of cancer, or during followup of cancer patients where normal tissue persists, such as in breast or colon cancer, the detection of these benign cells could be interpreted as a relapse of the primary cancer. This is in addition to the potential problem of CTCs that do not express EpCAM or cytokeratins [4]. 

The use of the biomarker P504S, although not prostate specific [5], has facilitated the differentiation between normal, dysplastic, and malignant tissues in prostate biopsy samples. Normal or benign cells do not express P504S, whereas cells arising from prostatic intraepithelial neoplasia (PIN) or cancer are positive [6]. Thus, at least in prostate cancer patients, the use of double immunomarcation could resolve this problem, whereby a malignant circulating prostate cell (CPC) would need to express both PSA and P504S, a benign CPC only PSA.

The prostate tumor early cancer test (ProTECT) study was started in 2008; the first stage evaluated 409 consecutive men attending a prostate cancer screening program in Chile. The thirty women acting as controls were all CPC negative; in the 409 men, the frequency of malignant CPC detection increased significantly with age and PSA level and is associated with a biopsy positive for cancer [7]. It was possible to detect malignant CPCs even at serum PSA levels of <2.0 ng/mL. The second stage was to determine the diagnostic yield of men with suspicion of prostate cancer because of an elevated serum PSA and/or abnormal DRE; 228 consecutive men undergoing prostate biopsy had a blood sample taken immediately beforehand to determine the presence or absence of malignant CPCs. The detection of malignant CPCs had a sensibility of 86.2%, specificity of 90.8%, a positive predictive value of 78.9%, and a negative predictive value of 94.3% [8]. It was noted that in some men without cancer detected on biopsy P504S negative, CPCs were detected.

In the present study, we analyze prospectively a cohort of patients with suspicion of prostate cancer based on an increased serum PSA and/or abnormal digital rectal examination and the presence of benign CPCs, defined as PSA positive P504S negative cells detected in venous blood and the results of the prostate biopsy.

## 2. Patients and Methods

The study was carried out between January 2009 and November 2012, in the Hospital de Carabineros de Chile (HOSCAR) and the Hospital de la Dirección de Prevision de Carabineros de Chile (DIPRECA). The immunocytochemistry was performed at the Instituto de Biooncology, Santiago, Chile. The study protocol and written consent forms were approved by the ethical committees of all the three centers. 

Consecutive men, aged between 45 and 80 years presenting to a prostate cancer screening program, without a previous history of prostate cancer or prostate biopsy and fulfilling the criteria a 12-core ultrasound guided transrectal PB were invited to participate. Biopsy criteria were serum PSA ≥4.0 ng/mL, serum PSA >0.75 ng/mL/year, and/or digital rectal examination (DRE) abnormal or suspicious of cancer. This was defined as the presence of a nodule, areas of indurations, or asymmetry in the size of the lateral lobes [9]. An ultrasound guided 12-core biopsy was taken according to standard recommendations [10].

Immediately before PB, an 8ml venous blood sample was taken in a tube containing EDTA (Beckinson-Vacutainer). Samples were maintained at 4°C and processed within 48 hours. The prostate biopsy and CPC detection were independently analyzed, with the evaluators being blinded to the clinical details and results of the biopsy or CPC test. 

## 3. Detection of CPCs

Mononuclear cells were obtained by differential centrifugation using Histopaque 1.077 (Sigma-Aldrich), washed and resuspended in 100 *μ*L of autologous plasma. 25 *μ*L aliquots was used to make slides (silianized, Dako, CA USA), dried in air for 24 hours, and fixed in a solution of 70% ethanol, 5% formaldehyde and 25% phosphate buffered saline pH 7.4.

CPCs were detected using a monoclonal antibody directed against PSA, clone 28A4 (Novocastra Laboratory, UK), and identified using an alkaline phosphatase-anti alkaline phosphatase-based system (LSAB2, DAKO, USA), with New Fuschin as the chromogen. Positive samples underwent a second process with anti-P504S clone 13H4 (DAKO, USA) and identified with a peroxidase-based system (LSAB2, DAKO, USA) with DAB (3,3′diaminobenzidine tetrahydrochloride) as the chromogen, according to the manufacturers' instructions.

**Figure 1:**
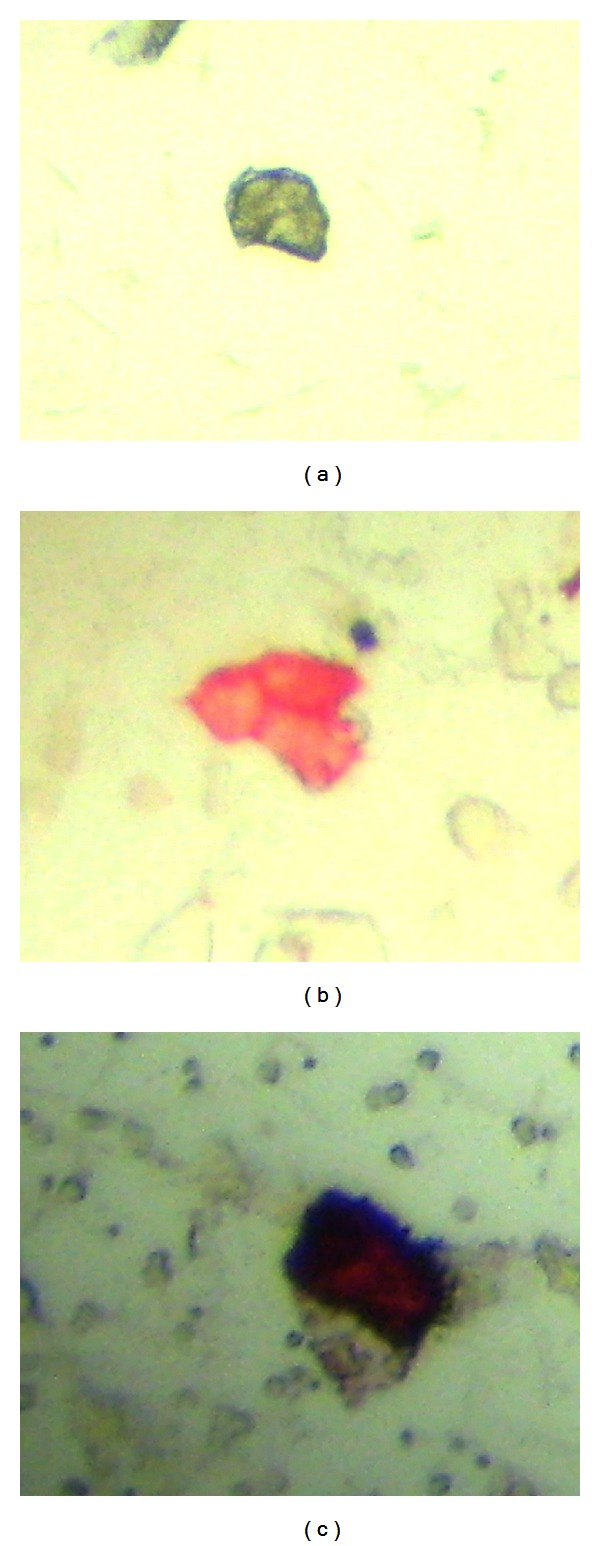
(a) Leucocyte PSA (−) P504S (−), (b) CPC PSA (+) P504S (−), and (c) CPC PSA (+) P504S (+).

A CPC was defined according to the criteria of (international society of hematotherapy and genetic engineering) ISHAGE [11] and the expression of P504S according to the consensus of the american association of pathologists [12]. A malignant CPC was defined as a cell that expressed PSA and P504S and a benign CPC as a cell that expressed PSA but not P504S, and leucocytes could be P504S positive or negative but did not express PSA (Figures 1(a)–1(c)). 

## 4. Statistical Analysis

The discrimination of the detection of benign CPCs was defined using the normal parameters: true positive (TP), false positive (FP), false negative (FN), and true negative (TN). The predictive values, positive (PPV), and negative (NPV) were evaluated, as well as the positive and negative likelihood ratios (+LR and −LR, resp.).

Descriptive statistics were used for demographic variables, expressed as mean and standard deviation in case of continuous variables with a normal distribution. In case of an asymmetrical distribution, the median and interquartile range (IQR) values were used. Noncontiguous variables were presented as frequencies. The Shapiro-Wilk test was used to determine a normal distribution. Student's *t*-test was used to compare continuous variables with a normal distribution, the Mann-Whitney test for ordinate and continuous variables with a nonnormal distribution, and Chi-squared for the differences in frequency. 

The diagnostic yield for the test-detecting benign CPCs was analyzed using standard parameters. For this purpose patients were classified as having or not having prostate cancer. For the purpose of the use of the number of mCPCs detected/mL as a diagnostic tool, and only as a mathematical exercise, the number of mCPCs/mL was considered as a continuous variable. Statistical significance was defined as a *P* value less than 0.05 to two sided. Analysis was performed using the Stata 11.0 program (StataCorp LP, College Station, TX, USA).

## 5. Results

359 men participated. The mean age was 65.6 ± 8.9 years and the median serum PSA was 5.20 ng/mL (Interquartile range (IQR) 4.31–7.30 ng/mL). 114/359 (31.8%) had prostate cancer detected on the initial biopsy. A total of 21(5.9%) men had CPCs P504S negative detected (Table 1).

The presence of P504S negative CPCs had a sensitivity of 9.29% (95% CI 5.85–13.85%) of detecting benign disease with a specificity of 100% (95% CI 96.78–100.00). The positive predictive value for benign disease was 100% (95% CI 83.75–100.00%) and the negative was predictive value 35.74% (95% CI 30.48–41.23%). The prevalence of benign disease (no cancer) was 61.67% (95% CI 61.68–71.47%).

Of the men with prostate cancer detected on biopsy, none had CPCs PSA (+) P504S (−) detected. The pathology report was defined as benign hyperplasia, benign hyperplasia with small foci of chronic prostatitis, or hyperplasia benign with chronic prostatitis. CPCs PSA (+) P504S (−) were associated with chronic prostatitis, with the frequency of benign CPCs being detected in 226 biopsies without evidence of prostate cancer, being 1/98 (1.0%) benign hyperplasia, 4/89 (4,5%) benign hyperplasia with small foci of chronic prostatitis, and 16/39 (41.0%) of men with benign hyperplasia with chronic prostatitis. Chi-squared for trends, *P* < 0.0001, with an overall risk of 1.00, 4.51, and 82.00, respectively.

## 6. Discussion

It has been considered that only cancer cells have the ability to disseminate or migrate into the circulation. However, these results and those published by Pantel et al. [3] suggest that benign inflammatory disease cells can escape into the circulation. The diagnosis of positively tested patients for PSA positive CPCs was based according to the strict criteria defined by ISHAGE with internal positive and negative controls. This finding is consistent with the fact that inflammatory cytokines can stimulate the migration of epithelial cells [13]. With respect to prostate cancer and the role of CPCs in its detection or in patients' pretreatment, the potential background of nonmalignant prostate cells in blood may be an important confounding factor and lead to false-positive findings in CPC diagnostics. The fact that these benign CPCs do not express P504S is important. In published studies using CPCs as a sequential test to detect prostate cancer [8], double immunostaining was used, only CPCs PSA (+) P504S (+) were considered to be malignant, whereas cells PSA (+) P504S (−) were considered to be benign and patients were classified as negative for cancer.

The results suggest that although P504S (−) CPCs are specific for benign disease, they are not sensitive in its detection; however, there is no association with the presence of a cancer.

This has implications on systems based on EpCAM, Cytokeratin, or PSA alone and may explain why no significant differences were found on the frequency of CPCs detected in early prostate cancer and controls [13–15]. Similarly using RT-PCR, 8% of patients with benign prostatic disease had CPCs detected [16].

In men after radical prostatectomy, there are no native prostate cells; thus all CPCs detected in blood have disseminated from metastatic microfoci and thus clinically represent cancer cells. This may explain why after primary surgery the use of EpCAM-, Cytokeratin- or PSA-based markers is associated with prognosis and survival [17]. 

However, after radiotherapy or brachytherapy with residual normal prostate tissue, this may not be the case. 

The fact that men with benign prostate cells detected did not have prostate cancer in the second or third biopsies decreases the possibility that some of these false-positive events were actually tumor cells arising from undetected cancer. However, in other tumors such as colon or breast where there is normal tissue present, this may cause clinical uncertainty as there is no marker such as P504S to differentiate between benign and malignant.

This study stresses the need that CPC detection, and by inference CTC detection used as a screening test or in patients with normal tissue remaining after primary treatment, should be further classified to determine their malignant state. In prostate patients, the use of double immunomarcation with P504S permits this differentiation and allows the test to be used in patients' pretreatment. In other cancers, the use of EpCAM- or cytokeratin-based methods does not permit this differentiation, given that most patients with benign inflammatory diseases have an excellent prognosis and will not develop cancer. 

In summary, patients with chronic prostatitis may have circulating prostate cells detected in blood, which do not express the enzyme P504S and should be thought of as benign in nature. There was no evidence that these patients had prostate cancer detected in subsequent biopsies. In other cancers, the presence of benign inflammatory tissue may cause migration of benign cells into the blood and thus cause false-positive findings.

## Figures and Tables

**Table 1:** The presence or absence of CPCs P504S negative and prostate biopsy results.

	Biopsy no cancer	Biopsy cancer	Total
CPC P504S (negative) present	21	0	21
CPC P504S (negative) absent	224	114	338

Total	245	114	359
